# Cytokine gene polymorphisms and atopic disease in two European cohorts. (ECRHS-Basel and SAPALDIA)

**DOI:** 10.1186/1476-7961-4-9

**Published:** 2006-06-07

**Authors:** M Imboden, A Nieters, AJ Bircher, M Brutsche, N Becker, M Wjst, U Ackermann-Liebrich, W Berger, NM Probst-Hensch, SAPALDIA Team

**Affiliations:** 1 Molecular Epidemiology/Cancer Registry, Institutes of Social and Preventive Medicine & Surgical Pathology, University Hospital Zurich, Switzerland; 2 Division of Medical Molecular Genetics and Gene Diagnostics, Institute of Medical Genetics, University of Zurich, Switzerland; 3 Division of Clinical Epidemiology, German Cancer Research Centre, Heidelberg, Germany; 4 Division of Allergology, University Hospital, Basel, Switzerland; 5 Departement of Pneumology, University Hospital, Basel, Switzerland; 6 GSF-National Research Center for Environment and Health, Institute of Epidemiology, Munich, Germany; 7 Institute of Social- und Preventive Medicine, University Basel, Switzerland

## Abstract

**Background:**

Atopy and allergic phenotypes are biologically characterized by an imbalanced T helper cell response skewed towards a type 2 (TH2) immune response associated with elevated serum immunoglobulin E (IgE) levels. Polymorphisms in cytokine genes might modulate regulation of the TH1/TH2 balance. We thus aimed at reproducing our previous findings from a European study population on the association of various cytokine polymorphisms with self-reported hay fever as well as increased total and specific IgE levels in two comparable study populations.

**Methods:**

Two prospective Caucasian cohorts were used. In the Basel center of the European Community Respiratory Health Survey (ECRHS, n = 418) ten distinct cytokine polymorphisms of putative functional relevance were genotyped. In the Swiss cohort Study on Air Pollution And Lung Disease In Adults (SAPALDIA, n = 6003) two cytokine polymorphisms were genotyped. The associations of these polymorphisms with atopy were estimated by covariance and logistic regression analysis.

**Results:**

We confirmed IL4, IL10, IL6 and IL18 as candidate genes for atopic health outcomes. In the large, well-characterized SAPALDIA cohort the IL6(-174G>C) and IL18(-137G>C) polymorphisms were associated with circulating total IgE concentrations in subjects with hay fever. The IL18(-137G>C) polymorphism was also associated with the prevalence of hay fever.

**Conclusion:**

Comprehensive characterization of genetic variation in extended cytokine candidate gene regions is now needed. Large study networks must follow to investigate the association of risk patterns defined by genetic predisposing and environmental risk factors with specific atopic phenotypes.

## Background

Atopic diseases like hay fever, asthma and eczema affect an increasing proportion of people and account for considerable morbidity and loss of quality of life. No simple pattern of inheritance has been shown and susceptibility to atopic disease appears to be determined by an interaction between environmental and genetic factors [1,2]. Cytokines have been shown to play a crucial role in the balance between TH1 and TH2 immune responses commonly thought to underlie atopic disease [3].

In recent years polymorphisms in various cytokine genes have been identified and indication of functional relevance exists for some of them. They have been associated with atopic disorders such as hay fever, asthma, eczema or elevated IgE levels, though inconsistently in many cases. We have previously investigated a combination of cytokine polymorphisms that we judged to have a high likelihood of functional relevance, with regard to risk of atopy and hay fever in a subsample of the European Prospective Investigation into Cancer and Nutrition (EPIC) [4]. A novel finding of our study was the association of a protective heterozygous effect of the IL6(-174C>G) polymorphism with the risk for hay fever and with IgE levels in hay fever cases.

We aimed to replicate our previously observed associations in two distinct study populations of European-Caucasian origin that were comparable to the EPIC study. The first study population is an asthma case over-sampled subpopulation of the European Community Respiratory Health Study (ECRHS)[5]. The second study population is a population-based cohort, the Swiss Study on Air Pollution and Lung Disease In Adults (SAPALDIA) [6,7]. The present research aims were i) to reproduce associations between ten potentially functionally relevant cytokine polymorphisms and atopic outcomes in the Basel ECRHS sample and ii) to investigate two of the least well established SNPs, IL6(-174G>C) and IL18(-137G>C) with increased statistical power in the SAPALDIA cohort.

## Materials and methods

### Study populations

One study population was the Swiss subsample of the European Community Respiratory Health Study (ECRHS). The European-wide cohort comprised at base line >10000 adult participants from 14 countries. Details of this pan-European cohort study have been reported elsewhere [5]. All participants of the Swiss ECRHS study center Basel who had given blood samples for IgE measurement and genotyping were included in the present study (n = 418). The second study population included in this paper is the Swiss Study on Air Pollution And Lung Disease In Adults (SAPALDIA) [6,7]. We included SAPALDIA participants with complete interview data, blood measurements of atopy at baseline, and available DNA samples for genotyping (n = 6003).

### IgE measurements

**ECRHS-Basel sample**: Total serum IgE levels and the concentrations of specific IgE to airborne allergens (cat, house dust mite, mold (*Cladosporium*) and timothy grass) were analyzed using the ELISA-based CAP system (Pharmacia Diagnostics, Uppsala, Sweden) [8]. Blood as well as interview data for this current study were collected at the ECRHS follow-up examination for the investigation of cross-sectional associations [9]. The measurement range for total IgE was 2 to 2000 kU/L and for specific IgE 0.35 to 100 kU/L. No measurements of total as well as specific IgE were obtainable from 21 ECRHS subjects of the Basel center. **SAPALDIA: **Circulating serum levels of total IgE and Phadiatop test were measured at baseline using the CAP FEIA system (Pharmacia Diagnostics, Uppsala, Sweden). Interview data was also obtained at baseline for cross-sectional analysis [6]. DNA for genotype analysis was extracted from blood samples collected at follow-up [7].

### Cases and controls

**ECRHS-Basel sample**: Irrespective of self-reporting of asthma or eczema, self-reported hay fever cases (n = 192) were defined by answering yes to the question: 'Have you ever had a problem with sneezing or a runny nose or a blocked nose when you did not have a cold or a flue?'. Irrespective of self-reporting of hay fever or eczema, physician-diagnosed asthma cases (n = 78) were defined by answering yes to both questions: 'Have you ever had asthma? Was this confirmed by a doctor?'. Irrespective of self-reporting asthma or hay fever, eczema cases (n = 200) were defined by answering yes to the question: 'Have you ever had eczema or any other kind of skin allergy?'. Participants who reported the absence of hay fever, eczema and asthma were defined as non-atopic controls (n = 125). Participants exhibiting total IgE levels higher than 100 kU/L were defined as "elevated total IgE cases". "Allergen sensitization cases" were defined by exhibiting at least one airborne allergen specific IgE higher than 0.35 kU/L. **SAPALDIA cohort**: The assessment of total IgE level and of various atopic disease outcomes in SAPALDIA was identical to the ECRHS study. In accordance with the definitions provided for ECRHS above, 1168 participants were defined as "elevated total IgE cases", 1105 participants as hay fever cases, 188 as asthma cases and 1837 as eczema cases. 2953 participants were identified as non-atopic controls. The "allergen sensitization cases" in the SAPALDIA cohort were defined by a positive result in the Phadiatop test (n = 1620).

### Cytokine genotypes

Genomic DNA was extracted manually using the Puregene^TM ^DNA Isolation Kit (Gentra Systems, Plymouth, MN, USA) for the ECRHS [9] and the SAPALDIA cohort [7]. In the ECRHS sample, RFLP and allele specific PCR was used for identification of the genetic polymorphisms as previously described [4]. Ten single nucleotide polymorphisms (SNP) were investigated in nine cytokine genes; SNP identification numbers (dbSNP:rs#) are listed in Table 2. Genotyping was conducted at the German Cancer Research Center under the supervision of one of us (AN). Genotyping failed in 8 samples for polymorphisms IL4R Q576R (A>G) and CD14(-159C>T), in 3 samples for IL6(-174G>C) and IL13 R130Q (A>G), in 2 samples for IL10(-819C>T) and TNF(-308G>A), in 1 sample for IL10(-1082G>A), IL12p40(1188A>C) and IL18(-137G>C). The considerably larger DNA sample collection of the SAPALDIA cohort (n>6000) were processed in a semi-automated medium throughput setup, assisted by liquid handling station (THEONYX, MWG, München, Germany) and subsequent 5'-nuclease fluorescent real-time PCR (TaqMan) genotyping assay was applied (Applera Europe, Rotkreuz, Switzerland). End-point detection was done using a 7000 ABI System detection device (ABI, Rotkreuz, Switzerland). Genotyping was conducted at the Institute of Medical Genetics, Zürich, under the supervision of one of us (MI). Genotyping failed in 16 samples for IL6(-174G>C) assay. Random re-genotyping of >5% of the samples showed a high reproducibility (>99.5%).

**Table 1:** Characteristics^1) ^of the study populations and genotype distribution.

	**ECRHS-Basel N = 418 (%)**	**SAPALDIA N = 6003 (%)**
Female proportion	211 (50.5)	3022 (50.3)
Age [years]	43.4 (± 7.0)	41.3 (± 11.4)
Age range [years]	29.3 to 55.1	18.2 to 61.8
Hay fever cases^2)^	192 (45.9)	1105 (18.4)
Asthma cases^3)^	78 (18.2)	369 (6.2)
Eczema cases^4)^	200 (47.9)	2383 (39.7)
Elevated total IgE cases^5)^	101 (24.9)	1168 (21.6)
Allergen sensitization cases^6)^	178 (42.6)	1620 (29.1)
Non-atopic controls^7)^	125 (29.9)	2953 (49.2)

^1) ^Presented in N (%) for categorical and in mean (± SD) for continuous variables.

^2) ^Self-reported hay fever cases answered yes to, Have you ever had a problem with sneezing or a runny nose or a blocked nose when you did not have a cold?', irrespective of self-reported asthma or eczema.

^3) ^Physician-diagnosed asthma cases answered twice yes to 'Have you ever had asthma? Was this confirmed by a doctor?', irrespective of self-reported hay fever or eczema.

^4) ^Self-reported eczema cases answered yes to 'Have you ever had eczema or any other kind of skin allergy?', irrespective of self-reported asthma or hay fever.

^5) ^Elevated total IgE cases had serum total IgE level >100 kU/L, measured by CAP system (Pharmacia)

^6) ^Allergen sensitization cases had at least one allergen specific IgE >0.35 kU/L, measured by CAP system (Pharmacia) for ECRHS-BS and by Phadiatop test (CAP FEIA system, Pharmacia) for SAPALDIA.

^7) ^Non-atopic controls were free of self-reported hay fever, self-reported eczema and physician-diagnosed asthma.

**Table 2:** Adjusted^1) ^associations of 10 cytokine polymorphisms with self-reported hay fever in the ECRHS-Basel Study.

**Genotype**		**cases/controls**^2)^	**Odds Ratio**	**95% Confidence Interval**	**P-value**
IL4 (-589C>T)	CC	140/89	1	--	--
rs2243250	CT	47/31	0.99	0.58 – 1.68	0.98
	TT	5/5	0.56	0.16 – 2.03	0.38
					
IL4R Q576R (A>G)	AA	128/71	1	--	--
rs1801275	AG	58/45	0.68	0.42 – 1.13	0.12
	GG	5/5	0.59	0.16 – 2.11	0.39
					
IL6(-174G>C)	GG	65/33	1	--	--
rs1800795	GC	97/66	0.77	0.45 – 1.30	0.32
	CC	28/25	0.58	0.29 – 1.15	0.12
					
IL10 (-1082G>A)	GG	60/48	1	--	--
rs1800896	GA	96/50	1.58	0.94 – 2.6	0.08
	AA	36/26	1.15	0.61 – 2.18	0.66
					
IL10 (-819C>T)	CC	102/71	1	--	--
rs1800871	CT	77/42	1.26	0.77 – 2.04	0.35
	TT	11/12	0.64	0.27 – 1.54	0.32
					
IL12p40(1188A>C)	AA	114/74	1	--	--
rs3212227	AC	69/44	0.99	0.61 – 1.59	0.95
	CC	9/6	1.05	0.36 – 3.09	0.93
					
IL13 R130Q (A>G)	GG	127/82	1	--	--
rs20541	GA	57/38	0.96	0.58 – 1.58	0.87
	AA	6/4	0.99	0.27 – 3.68	0.99
					
IL18(-137G>C)	GG	101/55	1	--	--
rs187238	GC	73/54	0.75	0.46 – 1.22	0.25
	CC	18/15	0.65	0.30 – 1.39	0.27
					
TNF(-308G>A)	GG	135/84	1	--	--
rs1800629	GA	53/35	0.94	0.57 – 1.57	0.83
	AA	4/4	0.58	0.14 – 2.40	0.45
					
CD14 (-159C>T)	CC	49/41	1	--	--
rs2569190	CT	89/55	1.36	0.79 – 2.32	0.26
	TT	51/26	1.64	0.88 – 3.09	0.12

^1) ^Adjusted for age and gender.

^2) ^Hay fever cases answered yes to 'Have you ever had a problem with sneezing or a runny nose or a blocked nose when you did not have a cold?', irrespective of self-reported asthma or eczema. Non-atopic controls were free of self-reported hay fever, self-reported eczema and physician-diagnosed asthma.

### Statistical analysis

Hardy-Weinberg equilibrium was tested using Arlequin Version 2.000 [10]. Genotype distribution for all cytokine SNPs was found to be in Hardy Weinberg equilibrium in both study populations. For the determination of an age- and sex-adjusted association between genotype and dichotomized phenotype (disease) we computed odds ratios (ORs), p-values and the corresponding 95% confidence limits (95%CI) using the STATA procedure LOGISTIC with dummy variables for the respective genotypes and with the most frequent allele as reference category. To assess the age-and sex-adjusted association between genotype and continuous phenotypes we computed adjusted means and p-values for group differences using the STATA procedure ONEWAY. Total IgE levels were log-transformed for analysis to achieve normal distribution. Adjusted means presented are geometric means. Statistical analysis was performed using STATA Version 8.1 SE (Stata Corporation, TX, USA). Two-sided P-values of <0.05 were considered as statistically significant. To correct for multiple comparison, we applied the Bonferroni correction (60 comparisons in the ECRHS sample and 28 comparisons in the SAPALDIA sample).

## Results

The two study populations were comparable with regard to the proportion of female participants and average age (Table 1). The ECRHS participants had a narrower age range and asthmatics had been over-sampled leading to increased proportions of hay fever, eczema cases, and atopy when compared to the SAPALDIA study cohort representative of the adult Swiss general population. We analyzed a panel of ten SNPs in nine different cytokine genes in the ECRHS-Basel study population. Two of these SNPs, IL18(-137G>C) and IL6(-174G>C), were also genotyped in the SAPALDIA study cohort.

The characteristics of the study populations are provided in Table 1. The age- and sex-adjusted association of each of the cytokine SNPs with self-reported hay fever in the Basel ECRHS study is presented in Table 2. None of the polymorphisms was associated with the prevalence of hay fever.

Age- and sex-adjusted associations of the cytokine SNPs with atopy, assessed by elevated total circulating IgE are presented in Table 3. Homo- or heterozygosity for the IL10(-819) T-allele was more prevalent among subjects with elevated total IgE levels than homozygosity for the C-allele (OR for CT genotype: 1.81; 95%CI 1.12–2.92 and OR for TT genotype: 1.96; 95%CI 0.82–4.67). The IL4(-589C>T) TT genotype was more prevalent among elevated total IgE cases (OR: 3.89; 95%CI 1.24–12.15). Homozygosity of the IL18(-137) C-allele was more prevalent among subjects with high total IgE levels compared to subjects with low total IgE levels (OR: 2.51; 95%CI 1.21–5.20) in the ECRHS-Basel sample.

**Table 3:** Adjusted^1) ^associations of 10 cytokine polymorphisms with elevated serum levels of total IgE in the ECRHS-Basel Study.

	**Elevated total IgE cases^2)^**				
**Genotype**		**cases/controls^3)^**	**Odds Ratio**	**95% Conficence Interval**	**P-value**
IL4 (-589C>T)	CC	69/227	1	--	--
	CT	25/81	1	0.57 – 1.65	0.91
	TT	7/6	3.89	1.24 – 12.15	0.02
					
IL4R Q576R (A>G)	AA	67/194	1	--	--
	AG	30/105	0.91	0.55 – 1.50	0.71
	GG	4/9	1.31	0.38 – 4.51	0.67
					
IL6(-174G>C)	GG	34/98	1	--	--
	GC	49/151	0.91	0.54 – 1.52	0.71
	CC	18/62	0.80	0.41 – 1.56	0.52
					
IL10 (-1082G>A)	GG	35/112	1	--	--
	GA	54/137	1.22	0.74 – 2.01	0.44
	AA	12/64	0.55	0.27 – 1.16	0.12
					
IL10 (-819C>T)	CC	44/180	1	--	--
	CT	48/112	1.81	1.12 – 2.92	0.02
	TT	9/20	1.96	0.82 – 4.67	0.13
					
IL12p40(1188A>C)	AA	61/183	1	--	--
	AC	39/112	1.03	0.64 – 1.66	0.89
	CC	1/18	0.16	0.02 – 1.20	0.08
					
IL13 R130Q (A>G)	GG	68/206	1	--	--
	GA	28/96	0.82	0.49 – 1.37	0.46
	AA	5/9	2.14	0.67 – 6.78	0.19
					
IL18(-137G>C)	GG	43/163	1	--	--
	GC	42/127	1.22	0.75 – 2.00	0.42
	CC	16/24	2.51	1.21 – 5.20	0.01
					
TNF(-308G>A)	GG	66/220	1	--	--
	GA	33/85	1.41	0.86 – 2.32	0.17
	AA	2/7	1.18	0.23 – 5.98	0.84
					
CD14 (-159C>T)	CC	24/88	1	--	--
	CT	55/140	1.44	0.83 – 2.52	0.19
	TT	21/79	0.97	0.50 – 1.90	0.94

^1) ^Adjusted for age and gender.

^2) ^Elevated total IgE cases had serum IgE level >100 kU/L, measured by CAP system (Pharmacia Diagnostics). Controls had IgE = 100 kU/L.

^3) ^Controls had IgE = 100 kU/L

We also analyzed the associations between cytokine SNPs and total circulating IgE levels, stratified by the presence of hay fever (data not shown). No statistically significant associations between any SNP and IgE levels were observed among subjects without atopic disease. Total IgE levels were elevated among participants with hay fever exhibiting IL4(-589C>T) TT genotype (p = 0.02). Hay fever cases exhibiting IL4R Q576R GG, IL10(-819) TT or IL18(-137) CC genotype had also increased total IgE levels, although not significant due to low statistically power.

Given the inconsistencies of the results observed for the two IL6 and IL18 SNPs when compared to our previous, novel finding from the EPIC substudy [4], we further investigated the role of these two SNPs in the larger SAPALDIA cohort.

In Tables 4, 5 and 6 we present the relationship of IL6 and IL18 SNPs with self-reported hay fever, and increased total IgE and specific allergen sensitization. For the IL18(-137G>C) SNP, we observed a statistically significant association with the risk of hay fever for heterozygous carriers compared to homozygous GG genotypes (OR: 1.24, 95% CI: 1.07 – 1.43, P = 0.004; Table 4). No other association of IL18 SNP with atopy biomarkers was observed. However among hay fever cases IL18(-137) CC genotype was associated with increased total IgE levels (P = 0.01; Table 6). For the IL6(-174G>C) SNP, no significant sex and age adjusted association was observed for questionnaire-based atopy reports, however for atopy biomarkers we observed an inverse association for heterozygotes of the IL6(-174G>C) with serum IgE levels >100 kU/L(OR: 0.83, 95% CI: 0.71–0.97, P = 0.02; Table 5). Subjects with IL6(-174) GG genotype exhibited higher total serum IgE levels than subjects with IL6(-174) GC or CC genotypes if they reported hay fever (Table 6). No statistically significant associations were observed of IL18 and IL6 genotypes with asthma or eczema (data not shown).

**Table 4:** Adjusted^1) ^association of IL6(-174G>C) and IL18(-137G>C) with hay fever^2) ^in the SAPALDIA Cohort Study.

		**Hay fever**			
**Genotype**		**cases/controls**^3)^	**Odds Ratio**	**95% Confidence Interval**	**P-value**
IL6(-174G>C)	GG	393/1112	1		--
	GC	537/1386	1.1	0.95 – 1.29	0.21
	CC	169/451	1.04	0.84 – 1.28	0.74
					
IL18(-137G>C)	GG	555/1619	1		--
	GC	468/1117	1.24	1.07 – 1.43	0.004
	CC	82/217	1.08	0.82 – 1.42	0.60

1) Adjusted for age and gender. ^2) ^Self-reported hay fever cases answered yes to 'Have you ever had a problem with sneezing or a runny nose or a blocked nose when you did not have a cold?', irrespective of self-reported asthma or eczema. ^3) ^Non-atopic controls were free of self-reported hay fever, self-reported eczema and physician-diagnosed asthma. ^4) ^Controls had total IgE ≤ 100 kU/L or were negative for all specific allergen tested (Phadiatop).

**Table 5:** Adjusted^1) ^association of IL6(-174G>C) and IL18(-137G>C) with elevated total IgE levels^2) ^and allergen sensitization^3)^in the SAPALDIA Cohort Study.

		**Elevated total IgE**				**Allergen sensitization (Phadiatop)**			
**Genotype**		**cases/controls**^4)^	**Odds Ratio**	**95% Confidence Interval**	**P-value**	**cases/controls**^4)^	**Odds Ratio**	**95% Confidence Interval**	**P-value**
IL6(-174G>C)	GG	462/804	1		--	596/1445	1		--
	GC	515/1078	0.83	0.71 – 0.97	0.02	768/1873	1	0.88 – 1.13	0.97
	CC	187/348	0.94	0.76 – 1.16	0.54	250/618	0.99	0.83 – 1.18	0.95
									
IL18(-137G>C)	GG	631/1237	1		--	860/2167	1		--
	GC	447/831	1.07	0.92 – 1.24	0.40	635/1504	1.1	0.95 – 1.21	0.26
	CC	90/164	1.07	0.82 – 1.41	0.61	125/275	1.1	0.91 – 1.43	0.27

1) Adjusted for age and gender. ^2) ^Elevated total IgE cases had serum levels of total IgE >100 kU/L, measured by CAP system (Pharmacia Diagnostics). ^3) ^Allergen sensitization cases had at least one positive allergen specific signal, measured by Phadiatop test using CAP FEIA system (Pharmacia Diagnostics). ^4) ^Controls had total IgE ≤ 100 kU/L or were negative for all specific allergen tested (Phadiatop).

**Table 6:** Mean adjusted^1) ^total serum IgE levels in cases and non-atopic controls^2) ^in dependence of IL6(-174G>C) or IL18(-137G>C) genotype in the SAPALDIA Cohort Study.

		**Hay fever**^3)^			**Controls**^2)^		
**Genotype**		**Number**	**Mean**^4)^	**P-value**	**Number**	**Mean**^4)^	**P-value**
IL6 (-174G>C)	GG	347	83.56		986	25.49	
	GC	483	61.29		1255	22.35	
	CC	148	62.26	0.009	412	23.29	0.11
							
IL18 (-137G>C)	GG	494	71.85		1464	23.79	
	GC	419	60.73		997	23.21	
	CC	71	103.20	0.01	196	24.96	0.80

1) Adjusted for age and gender. ^2) ^Non-atopic controls were free of self-reported hay fever, self-reported eczema and physician-diagnosed asthma. ^3) ^Self-reported hay fever cases answered yes to 'Have you ever had a problem with sneezing or a runny nose or a blocked nose when you did not have a cold?', irrespective of self-reported asthma or eczema. ^4) ^Measured total serum IgE levels were log-transformed and adjusted means are geometric means.

## Discussion

Our results from the ECRHRS Basel and the SAPALDIA studies confirmed previously reported associations of genetic variation in IL4, IL10, and IL18 cytokine genes with atopic phenotypes. In addition, in the large SAPALDIA cohort we were able to confirm the previously reported, novel association between the IL6(-174G>C) genotype and atopic phenotypes. Homozygosity for the G-allele was associated with increased total serum IgE concentrations in subjects reporting hay fever.

IL4 and IL10 has long been investigated as potential candidate genes for asthma and atopy [11]. IL4 is a pleiotrophic TH2 cytokine and impacts on the development of asthma and atopy in part through its role in the differentiation to a TH2 phenotype of T cells. Moreover IL4 is responsible for the class-switching from IgM to IgE. Genetic variation in the IL4 gene has shown linkage to atopy and asthma in several studies; common promoter polymorphisms including the IL4(-589C>T) SNP have been associated with asthma and/or atopy in many studies [12]. IL10 is an anti-inflammatory cytokine that suppresses the TH1-response and promotes B-cell activation as well as regulates immunoglobulin class switching. According to *in vitro *tests, IL10 regulates IgE production and reduces IgE switching in the presence of IL4. Various SNPs and haplotypes in the IL10 have been associated with atopic phenotypes including circulating IgE concentrations [13].

The observed associations between the IL18 SNP and hay fever or atopy are consistent with IL18 being a determinant of TH1 and TH2 differentiation. IL18 has been suggested to play a pleiotrophic role in the TH1/TH2 balance [14,15]. Recent evidence suggests that IL18 and genetic variation in this gene are associated with atopy [16-19] and asthma [19-22]. The SNP investigated has been shown to be functionally relevant in vitro; the position (-137) of the IL18 promoter is part of the binding site for nuclear transcription factors [23]. Depending on the presence of a G- or C-nucleotide at this polymorphic site, different transcription factors have been suggested to recognize it and thus might differentially activate the gene.

We were able to confirm our previously reported, novel finding of an association between IL6 genotype and atopic phenotypes by observing elevated IgE concentrations among G/G genotypes. Contrary to our findings from the EPIC cohort, we could not replicate the association with the prevalence of hay fever in the large SAPALDIA cohort, though [4]. Increasing evidence implicates IL6 in promoting the development of TH2 mediated diseases, like allergies (reviewed in [4]). In addition, this cytokine and its genetic variants have more commonly been associated with pro-inflammatory and specifically with acute phase inflammation states. Indirect involvement of IL6 in the etiology of environmentally induced atopy and development of asthma can therefore not be excluded. For example it is known that air pollution exposure acts through oxidative stress promoting inflammatory processes and thus might increase the risk of atopic airway exacerbations and disease development [24]. Circulating IL6 concentrations have been shown to be increased in children exposed to air pollution [25].

This current study has several strong aspects. First, we pursued a focused candidate gene approach. Our primary goal was the replication of findings from our previous study in two comparable populations. Our previous study already focused on polymorphisms with a strong prior hypothesis for potential association with atopy based on both, previous reports from association studies as well as functional studies of the SNPs [4]. Second, our results are in line with well established associations of SNPs in IL4(-589C>T) [11,12,26,27] and IL10 [11,27] with asthma [11,12,28] and atopy [26,27,29]. We also observed a positive association of CD14(-159C>T) with asthma (data not shown), an association that has also previously been observed [28]. These results support the validity of the results obtained from the ECRHS-Basel sample despite its restricted sample size. Third, the extensive sample size and detailed characterization of the SAPALDIA cohort study provided a setting to corroborate with sufficient statistical power the potential role of genetic variation in IL18 and even more importantly in IL6 in the etiology and progression of atopic diseases.

A limitation of the ECRHS part of the study was the small sample size. Accordingly, none of the statistically significant results withheld the conservative correction for multiple testing. Applying the Bonferroni correction to the ECRHS results lead to a revised significance level of 0.0008 (60 comparisons) and according corrections to the results obtained in SAPALDIA study lead to a revised significance level of 0.0017 (28 comparisons). We chose to present the uncorrected results because a) several of the statistical tests performed were not independent, and b) all association tested reflected an a priori hypothesis of the study.

The rather low reproducibility of the observed associations for additional SNPs across the three studies is consistent, though, with the generally poor reproducibility of many genotype-disease associations [30]. Likely explanations for the lack of replication include insufficient power, population stratification and differences in linkage disequilibrium between study population [30], chance findings and publication bias, as well as heterogeneity in genetic and environmental modifiers of specific gene/disease associations. We evaluated here only a small number of selected genes and of specific SNP, yet other genes and more importantly other sites of genetic variations should be explored in future association studies. Of specific relevance to the future investigation of genetic variation in cytokine genes is the comprehensive identification of genetic variation and common haplotypes in the according gene regions [12, 31]. This seems of special relevance since many of the cytokine genes are found in chromosomal clusters [31]. Genetic variants within and between different cytokine genes are therefore likely to be in strong linkage disequilibrium.

## Conclusion

The results of this replication study further establish IL4, IL10, IL18 and IL6 as candidate genes for atopic health outcomes. Future networks of studies must now focus on comprehensively characterizing genetic variation in extended regions of these cytokine genes. The investigation of gene-gene-interactions seems essential given our understanding of the complex interplay between various cytokines in each others regulation. Finally, potential modification of genotype and haplotype effects by environmental and lifestyle factors and specific disease phenotypes will further help to clarify our understanding of atopic disease.

## Authors' contributions

NP and AN conceived and designed the study. MW carried out the DNA extraction of the ECRHS population. AN and NB carried out the molecular genetic analysis on the ECHRS-Basel subsample. MI, WB and NP carried out the DNA extraction and genotyping analysis of the SAPALDIA cohort. MI and NP performed association analysis on both study population and drafted the manuscript. MB, was involved in the examination of the SAPALDIA probands. MW, AN, NB, MB, AJB and UA contributed to the interpretation of results and the manuscript. All authors read and approved the final manuscript.
